# Analysis of Minor Proteins Present in Breast Milk by Using WGA Lectin

**DOI:** 10.3390/children9071084

**Published:** 2022-07-20

**Authors:** Iván Hernández-Caravaca, Andrés Cabañas, Rebeca López-Úbeda, Leopoldo González-Brusi, Ascensión Guillén-Martínez, Mª José Izquierdo-Rico, Mª Nieves Muñoz-Rodríguez, Manuel Avilés, Mª Jesús Ruiz García

**Affiliations:** 1 Department of Community Nursing, Preventive Medicine and Public Health and History of Science, University of Alicante, Sant Vicent del Raspeig, 03690 Alicante, Spain; 2 IMIB-Arrixaca (Instituto Murciano de Investigación Biosanitaria), 30120 Murcia, Spain; rebeca_rlu@hotmail.com (R.L.-Ú.); leopoldo.gonzalez@um.es (L.G.-B.); ascension.guillen@um.es (A.G.-M.); mjoseir@um.es (M.J.I.-R.); maviles@um.es (M.A.); 3 Department of Cell Biology and Histology, Faculty of Medicine and Nursing, University of Murcia, Campus Mare Nostrum, 30100 Murcia, Spain; andres.cabanas@um.es; 4 Coordinación Sociosanitaria Dirección General de Planificación, Investigación, Farmacia y Atención al Ciudadano (CARM), 30071 Murcia, Spain; mnieves.munoz@carm.es; 5 Department of Nursing, Faculty of Nursing, University of Murcia, Campus Mare Nostrum, 30100 Murcia, Spain; mjruiz@um.es

**Keywords:** breast milk, proteomics, glycosylated proteins, WGA lectin, minor proteins

## Abstract

Breast milk is a complex and dynamic biological fluid and considered an essential source of nutrition in early life. In its composition, the proteins have a relevant biological activity and are related to the multiple benefits demonstrated when compared with artificial milks derived from cow’s milk. Understanding human milk composition provides an important tool for health care providers toward the management of infant feeding and the establishment of breastfeeding. In this work, a new technique was developed to increase the knowledge of human milk, because many of the components remain unknown. To isolate minor proteins present in breast milk by using WGA lectin, breast milk was centrifuged to remove cells and separate the fat phase from the serum phase. The serum obtained was separated into two groups: control (*n* = 3; whole serum sample from mature milk) and WGA lectin (*n* = 3; sample processed with WGA lectin to isolate glycosylated proteins). The samples were analyzed by high-performance liquid chromatography coupled to mass spectrometry (HPLC/MS). A total of 84 different proteins were identified from all of the samples. In the WGA lectin group, 55 different proteins were isolated, 77% of which had biological functions related to the immune response. Of these proteins, there were eight WGA lectin group exclusives, and two had not previously been described in breast milk (polyubiquitin-B and POTE ankyrin domain family member F). Isolation by WGA lectin is a useful technique to detect minor proteins in breast milk and to identify proteins that could not be observed in whole serum.

## 1. Introduction

Breast milk is the most important food for the new-born [1], as it is unique in terms of its nutritional composition, immune system components, anti-infective factors and metabolic enzymes. It is considered the best source of infant nutrition, and is recommended by the World Health Organization as the sole source of food during the first six months of life, and up to at least two years of age as a complementary food [2,3]. Many studies have shown how breastfeeding is associated with a low incidence of obesity, diabetes and cardiovascular disease throughout an individual’s life [4,5,6,7]. Moreover, it is known that it reduces the risk of infectious diseases and non-communicable diseases such as asthma, cancer and autoimmune diseases [8]. The benefit of breastfeeding for mothers is also demonstrated in several studies that show that there is an inverse relationship between the duration of breastfeeding and breast and ovarian cancer [9,10,11].

Breast milk has also been shown to be a dynamic product, with different characteristics depending on the time of production, and, with a decreasing protein content, the duration of breastfeeding increases. Colostrum is the type of milk that has a higher protein content, followed by transition milk and, finally, mature milk, with a lower amount of protein [8]. However, many of the components of this complex food remain unknown.

Among the benefits provided to infants, the protein content of breast milk plays an important role. Proteins represent 50–80% of the components of the whey fraction of the milk, whereas the proteins found in the casein micelles are a smaller fraction. These proportions of the two types of protein vary with the stage of lactation [12,13,14], and it is also possible to find proteins, but in smaller proportions, in other cellular fractions and in fat globules [15,16]. 

Many of these proteins are resistant to proteolysis, which, together with a developing neonatal digestive system, facilitates the survival of biologically active forms of milk protein in the infant gastrointestinal tract [17,18]. It has been described how IgA from serum prevents infection of the intestinal mucosa by binding microorganisms and preventing them from being transported through the mucosa [19,20], just as it modulates the function and integrity of the gastrointestinal tract [21,22,23]. In addition to the direct effect of these proteins, the peptides released during enzymatic digestion have their own biological activity and can act as growth factors, neurotransmitters and hormone inducers in vasoregulation [24,25]. Other functions of breast milk proteins include a wide variety of bioactivities, such as immunomodulatory, bacteriostatic, prebiotic (along with oligosaccharides), bactericidal, antiapoptotic, proteolytic and lipolytic activities, and they also collaborate in organ maturation, including brain development and the maturation of the immune system and digestive tract [26,27,28].

Many of the proteins present in breast milk are glycosylated [29,30,31,32], a process that has been shown to play a key role in many of the biological functions of proteins, stabilizing their structure, mediating cell signalling and recognition and modulating adhesion and invasion during infection [31,33].

Although our knowledge about the protein composition of breast milk and its biology is wide in terms of publications, there are still opportunities regarding the detection of minor proteins and their function with the use of new techniques. Understanding which new components are involved in human milk and their functions will lead to progress in child nutrition and enable improvements to be made to currently used infant formulas. This work aimed to characterize the minor components of breast milk using a new methodology—mass spectrometry after isolation by WGA lectin—by means of which the glycosylated proteins present in breast milk can be detected.

## 2. Materials and Methods

### 2.1. Ethics

This study was approved by the Research Ethical Committee of the University of Murcia, Spain. A written informed consent was obtained from all donors involved in this study.

### 2.2. Collection and Processing of Samples

Breast milk samples were manually collected from three healthy donors into sterile polypropylene containers. The donors were selected to represent different characteristics with regard to the number of births, maternal age and time of lactation (donor 1: primiparous, 30 years, 5 months of breastfeeding; donor 2: multiparous, 33 years, 12 months of breastfeeding; and donor 3: multiparous, 41 years, 18 months of breastfeeding). The samples were kept at 4 °C during transport to the laboratory to avoid proteolysis, and were kept at −80 °C until analysis. 

### 2.3. Protein Extraction and Isolation Using WGA Lectin

For this purpose, 15 mL of each of the milk samples were centrifuged at 15,928× *g* for 30 min at 4 °C to remove the cells and separate the fat and serum phases. The volume of serum obtained from each of the samples was separated into two groups. The control group (*n* = 3; whole serum sample) was stored at 4 °C for later analysis, whereas the rest of the sample was processed to isolate the glycosylated proteins using WGA lectin (*n* = 3; WGA lectin sample). The WGA lectin from *Triticum vulgaris* (L1882; Sigma-Aldrich, Madrid, Spain) was used to purify the serum glycoproteins. For each sample, 100 μL of WGA lectin was washed twice with 500 μL phosphate-buffered saline (PBS) (24× *g* for 30 s at room temperature). Once the lectin had precipitated, 50 μL of serum and 400 μL of PBS were added, and the mixture was resuspended by shaking gently for 45 min. The mixture was then centrifuged for 30 s at 24× *g*, before removing the supernatant and washing twice with 600 μL of PBS. After removing the supernatant, the samples were stored at −80 °C until analysis.

### 2.4. Peptide Fragmentation by Trypsin

All samples (*n* = 6) were diluted in 100 μL of 50 mM ammonium bicarbonate buffer pH 8.5 with 0.01% ProteaseMax (Promega, Madison, WI, USA) and 20 mM DTT, and incubated for 20 min at 56 °C. After this process, the samples were blocked by adding 100 mM iodoacetamide and incubated for 30 min at room temperature in the dark. Finally, the samples were digested by adding 1 μg trypsin (Trypsin Gold Mass Spectrometry Grade (V5280), Promega, Madison, WI, USA) for 3 h at 37 °C. The reaction was stopped with 0.1% formic acid and filtered through a 0.2 μm pore filter. The samples were dried using a vacuum concentrator (Model 5301, Eppendorf, Hamburg, Germany).

### 2.5. Separation by High-Performance Liquid Chromatography Coupled to Mass Spectrometry (HPLC/MS)

Separation and analysis of the tryptic digestions of the samples were carried out by high-resolution liquid chromatography coupled to mass spectrometry (HPLC/MS), using an Agilent Model 1100 Series HPLC, thermostated and equipped with an automatic sampler and capillary pump. This HPLC was connected to an Agilent XCT Plus ion trap mass spectrometer by means of an electrospray interface (ESI). Previously digested and evaporated samples were resuspended in 20 μL of buffer A consisting of a water/acetonitrile/formic acid mixture (94.9:5:0.1). In a thermostatically controlled compartment at 40 °C, the sample was injected into a Waters XBridge BEH C 18 HPLC column for peptide separation and analysis at a flow rate of 10 μL/min. After injection, the column was washed with buffer A, and the digested peptides were separated using a linear gradient of 0–80% buffer B lasting 150 min. Buffer B consisted of a water/acetonitrile/formic acid mixture (10:89.9:0.1). The mass spectrometer was used in positive mode, with a capillary voltage of 3500 V. The MS/MS data were collected automatically. The strongest ions were fragmented sequentially by collision-induced dissociation (CID) using helium as the collision gas.

### 2.6. Bioinformatics Analysis and Identification of Proteins

Data were processed with the LC/MSD Trap Data Analysis Version 3.3 program (Bruker Daltonik, GmbH, Bremen, Germany), and the search for matches was performed with the Spectrum Mill engine (Agilent Technologies, USA) against the Uniprot human protein database. For validation of peptides and proteins, we followed the indications of Spectrum Mill software using auto-threshold validation. To detect glycoproteins with terminal sialic acid (a WGA lectin ligand) on their N- or O-linked glycans, Uniprot IDs were queried with an application programming interface (API) customized for GlyConnect [34]. The gene ontology of the proteins detected by WGA was evaluated with the DAVID program (https://david.ncifcrf.gov/summary.jsp, accessed on 15 January 2022), which determines the biological processes in which they are involved. This program was also used when the data obtained with Glyconnect were not sufficient to confirm the presence of sialic acid in the glycans of the proteins in order to determine if they had been previously identified as N-glycoproteins.

## 3. Results

**Figure 1:**
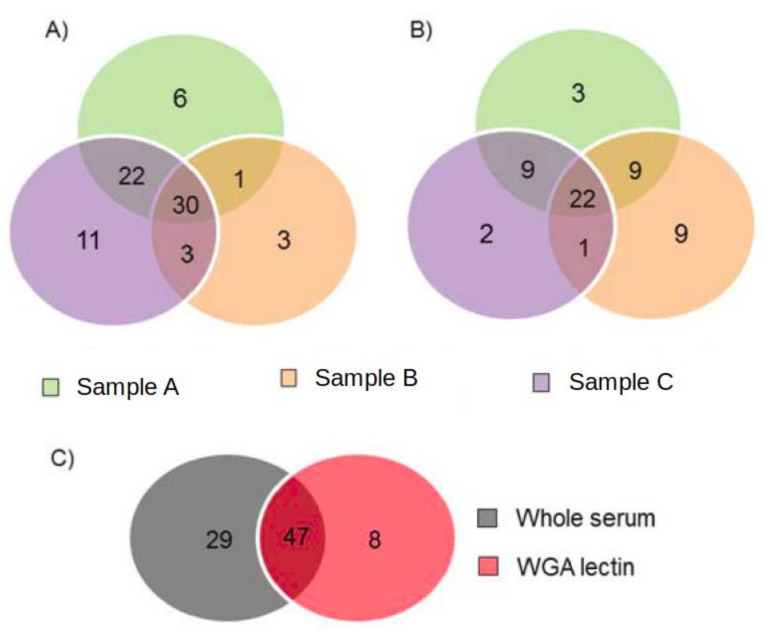
Venn diagram comparison of the identified proteins. (**A**) Whole serum samples. (**B**) Samples processed by WGA lectin. (**C**) Serum samples vs. samples processed by WGA lectin.

Taking into account all of the samples (*n* = 6), a total of 84 different proteins were identified. The details of all the proteins identified in each of the samples are summarized in Supplementary Table S1. A total of 76 different proteins were found in the analysis of the whole serum samples (*n* = 3), of which, 30 were common in all of the whole serum samples analyzed (Figure 1A); that is, 39.5% of the proteins were detected in all samples. A total of 55 different proteins were identified in the study of the samples treated with WGA lectin (*n* = 3), of which, 22 (i.e., 40%) were detected in all of the samples analyzed using this technique (Figure 1B).

**Figure 2:**
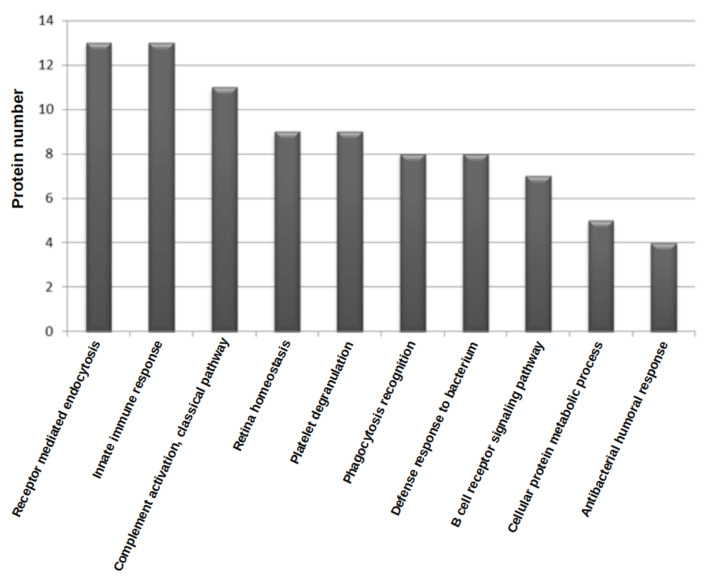
Biological processes involving the proteins detected in the WGA lectin group.

Of the 55 proteins detected by WGA lectin, 82% (45 proteins) can be described as potential WGA ligands because they were N-glycoproteins or proteins with sialic acid as the terminal sugar. By studying the biological processes of the proteins from the WGA treatment group (Figure 2), it was seen that 77% of the functions are related to the immune response (13 proteins related to innate immune response, 11 to complement activation, 8 to phagocytosis and 8 to antibacterial response).

**Figure 3:**
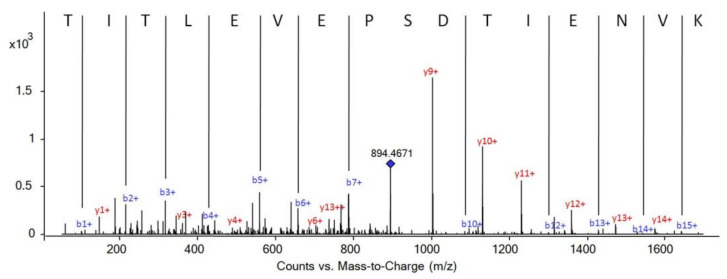
ESI-MS/MS spectra of Polyubiquitin-B peptide 12–27 (TITLEVEPSDTIENVK). Score 15.59, SPI 87.4%, 894.4671 *m*/*z* (shown with a blue diamond) (*z* = 2). The **y** ions are shown in red and the **b** ions in blue.

When the proteins of the different treatments were compared (whole serum; *n* = 76 proteins and WGA lectin treatment; *n* = 55), it was observed that 47 of the proteins were common and were detected in both groups (Figure 1C), with 17 of them appearing in all of the samples analyzed. On the other hand, 29 proteins were only detected in serum; that is, they did not appear in any sample treated with WGA lectin. In the WGA lectin group, eight of the identified proteins were not detected in the serum samples (alpha-1-acid glycoprotein 1, alpha-amylase 1, E3 ubiquitin-protein ligase MYCBP2, immunoglobulin heavy variable 3/OR16-9, immunoglobulin kappa variable 3–20, immunoglobulin kappa variable 3D-7, polyubiquitin-B and POTE ankyrin domain family member F) (Table 1). ESI-MS/MS spectra of a peptide corresponding to Polyubiquitin-B are shown in Figure 3.

## 4. Discussion

Previous studies have demonstrated the wide variety of protein components that make up breast milk [35,36,37], as well as how their concentration and composition vary during the maturation of the milk [31,37,38]. It is known that the major protein components of breast milk are caseins, milk fat globule membrane (MFGM) proteins and predominant whey proteins [31,35]. These proteins make it difficult to detect other proteins that are present in lower proportions. These minor proteins could have important biological activities and their analysis could be useful for understanding the complete composition of maternal milk. Therefore, the aim of this work was to detect minor protein components in milk using WGA lectin. 

A total of 84 different serum proteins were identified by HPLC/MS. In the samples treated with WGA lectin, a total of 55 proteins were identified, 45 of them corresponding to N-glycosylated proteins or to sialic acid present in the form of terminal sugars, which is necessary for them to be detected by WGA lectin, confirming that isolation by WGA lectin is a valid technique for detecting minor proteins. Of the proteins detected, eight were exclusive to this treatment and can be divided into two groups: proteins previously described in breast milk and those that have not previously been described.

Among the previously described proteins was alpha-1-acid glycoprotein 1, which is synthesized by human mammary epithelial cells [39] and whose presence has been described both in human breast milk [40,41] and in cow colostrum [42]. Although its function has not been clearly defined, it has been suggested that it may influence the effectiveness of the defense mechanisms of innate immunity in the newborns [43], perhaps by inhibiting the adhesion of sialic-acid-dependent pathogens that cause severe gastroenteritis [44]. Another protein detected was alpha-amylase 1, which has been described previously in breast milk [45]. This is a key enzyme in the digestion of starch and polysaccharides and its main function would seem to be to compensate for the low activity of the salivary gland and pancreas in newborns, especially premature babies. In the case of women, their presence has been detected in both milk and colostrum [46], and it has been shown that this protein is locally secreted by the mammary gland [47]. Finally, three of the proteins described above are included in the family of immunoglobulins (immunoglobulin heavy variable 3/OR16-9, immunoglobulin kappa variable 3–20, immunoglobulin kappa variable 3D-7), which are known to be present in breast milk and which form an essential part of the passive immunity passed from mother to offspring in order to promote the development of the immune system [48].

The remaining proteins detected by WGA lectin have not previously been described in breast milk: polyubiquitin-B and the POTE ankyrin domain family member F (POTE F). The POTE F protein belongs to the POTE gene family and its expression has been detected in several tissues, such as prostate, ovary, testicles and placenta [49], as well as in several organs and tissues with cancer, such as the mammary gland, colon, prostate or ovaries [50,51]. Recent studies have shown that the POTE E or POTE F protein has a pro-apoptotic function in vitro [52]. To date, few previous studies have reported the in vivo functions of the POTE gene family. A suggested in vivo function in ovarium is that a proper amount of POTE F is required for the maintenance of granulosa cells (GC) in follicle pools, whereas POTE F overaccumulation might be involved in follicle atresia and the development of primary ovarian insufficiency. Notably, POTE F is detected in GCs of primordial follicles and primary follicles, and hardly observed in growing secondary, preantral and small antral follicles. These findings suggest that POTE F could contribute to the growth of GCs in early follicles, which might be involved in the regulation of single ovulation, specifically in primates [53]. Moreover, another important study has POTE F as a relevant marker of cancer metastases. Altered glycans or the aberrant protein glycosylation of POTE F on cell surfaces was related to the breast cancer with the worst overall survival, the triple-negative breast cancer (TNBC). The authors suggest that POTE F could be involved in metastatic capacities such as cell invasion, migration and adhesion. In our study, we have detected for the first time POTE F in breast milk. Further studies can be conducted to determine whether this altered POTE F in TNBC can also be detected in breast milk, helping to diagnose this kind of cancer during the breastfeeding [54]. 

The function of polyubiquitin B (UBB) in milk is difficult to study as it would require a conditional knockout of the mammary gland, due to the animals being sterile [55]. However, UBB is a protein that has been linked to neurogenesis and neuronal maturation [56,57]. It has been demonstrated that the disruption of the polyubiquitin gene homeostasis affects the self-renewal of neural stem cells, resulting in altered neuronal differentiation in polyubiquitin B knockout mice [58]. Thus, its presence in breast milk and the maintenance of breastfeeding could promote a proper neuronal development of the baby. In addition, UBB is involved in maintaining the nuclear chromatin structure, and is related to the response to cellular stress and protein degradation [59]. Moreover, it can be found in exosomes of multiple body fluids, and the ubiquitin–proteasome system mediates the effect of glucose on milk fat synthesis in cows [60]. Therefore, ubiquitin may be a potential biomarker of milk quality. However, this is the first description of these two proteins in breast milk, and further studies will be necessary to determine their specific function in breast milk.

## 5. Conclusions

Our findings in the present study demonstrate that the use of WGA lectin is a useful technique for detecting minor breast milk proteins. WGA lectin analysis detected eight proteins not observed in the control group. The biological processes of 77% of the proteins detected by WGA are involved the immune response. Further, two proteins not previously described in breast milk (POTE F and polyubiquitin B) were identified using this method. Moreover, these new discoveries about human milk composition provide new arguments for breastfeeding promotion. 

## Figures and Tables

**Table 1:** Proteins detected exclusively in the samples treated with WGA lectin.

Access Number *	Protein Name	Outlined Above
P02763	Alpha-1-acid glycoprotein 1	√
P04745	Alpha-amylase 1	√
O75592	E3 ubiquitin-protein ligase MYCBP2	√
A0A0B4J2B5	Immunoglobulin heavy variable 3/OR16-9	√
P01619	Immunoglobulin kappa variable 3–20	√
A0A075B6H7	Immunoglobulin kappa variable 3D-7	√
B4DV12	Polyubiquitin-B	
A5A3E0	POTE ankyrin domain family member F	

* Unique identifier assigned to each protein in the UniProt database.

## Data Availability

Not applicable.

## Supplementary Materials

The following supporting information can be downloaded at: https://www.mdpi.com/article/10.3390/children9071084/s1, Supplementary Table S1: Proteins identified by mass spectrometry in each of the samples.

## References

[1] Lessen R., Kavanagh K. (2015). Position of the academy of nutrition and dietetics: Promoting and supporting breastfeeding. J. Acad. Nutr. Diet..

[2] (2002). Assembly. WHOF-fWH. Infant and Young Child Nutrition: Globalstrategy on Infant and Young Child Feeding Published. http://apps.who.int/gb/archive/pdf_files/WHA55/ea5515.pdf.

[3] Eidelman A.I., Schanler R.J. (2012). Breastfeeding and the use of human milk. Pediatrics.

[4] Koletzko B., von Kries R., Monasterolo R.C., Subias J.E., Scaglioni S., Giovannini M., Beyer J., Demmelmair H., Anton B., Gruszfeld D. (2009). Infant Feeding and Later Obesity Risk. Early Nutrition Programming and Health Outcomes in Later Life.

[5] Martin R.M., Gunnell D., Davey Smith G. (2005). Breastfeeding in infancy and blood pressure in later life: Systematic review and meta-analysis. Am. J. Epidemiol..

[6] Owen C.G., Whincup P.H., Kaye S.J., Martin R.M., Smith G.D., Cook D.G., Bergstrom E., Black S., Wadsworth M.E.J., Fall C.H. (2008). Does initial breastfeeding lead to lower blood cholesterol in adult life? A quantitative review of the evidence. Am. J. Clin. Nutr..

[7] Savino F., Benetti S., Liguori S.A., Sorrenti M., Cordero Di Montezemolo L. (2013). Advances on human milk hormones and protection against obesity. Cell. Mol. Biol..

[8] Lokossou G.A.G., Kouakanou L., Schumacher A., Zenclussen A.C. (2022). Human Breast Milk: From Food to Active Immune Response with Disease Protection in Infants and Mothers. Front. Immunol..

[9] Collaborative Group on Hormonal Factors in Breast Cancer (2002). Breast Cancer and Breastfeeding: Collaborative Reanalysis of Individual Data From 47 Epidemiological Studies in 30 Countries, Including 50,302 Women with Breast Cancer and 96,973 Women Without the Disease. Lancet.

[10] Chowdhury R., Sinha B., Sankar M.J., Taneja S., Bhandari N., Rollins N., Bahl R., Martines J.C. (2015). Breastfeeding and Maternal Health Outcomes: A Systematic Review and Meta-Analysis. Acta Paediatr..

[11] Victora C.G., Bahl R., Barros A.J.D., Franca G.V.A., Horton S., Krasevec J., Murch S., Sankar M.J., Walker N., Rollins N.C. (2016). Breastfeeding in the 21st Century: Epidemiology, Mechanisms, and Lifelong Effect. Lancet.

[12] Lönnerdal B., Forsum E., Hambraeus L. (1976). A longitudinal study of the protein, nitrogen, and lactose contents of human milk from Swedish well-nourished mothers. Am. J. Clin. Nutr..

[13] Guo M. (2014). Human Milk Biochemistry and Infant Formula Manufacturing Technology.

[14] Liao Y., Weber D., Xu W., Durbin-Johnson B.P., Phinney B.S., Lönnerdal B. (2017). Absolute quantification of human milk caseins and the whey/casein ratio during the first year of lactation. J. Proteome Res..

[15] Lönnerdal B. (2003). Nutritional and physiologic significance of human milk proteins. Am. J. Clin. Nutr..

[16] Kelleher S.L., Lönnerdal B. (2001). Immunological Activities Associated with Milk. Advances in Nutritional Research.

[17] Beverly R.L., Huston R.K., Markell A.M., McCulley E.A., Martin R.L., Dallas D.C. (2019). Milk Peptides Survive In Vivo Gastrointestinal Digestion and Are Excreted in the Stool of Infants. J. Nutr..

[18] Picariello G., Ferranti P., Fierro O., Mamone G., Caira S., Di Luccia A., Monica S., Addeo F. (2010). Peptides surviving the simulated gastrointestinal digestion of milk proteins: Biological and toxicological implications. J. Chromatogr. B.

[19] Hanson L.Å., Korotkova M., Telemo E. (2005). Human milk: Its Components and their Immunobiologic Functions. Mucosal Immunology.

[20] Russell M.W. (2007). Biological Functions of IgA. Mucosal Immune Defense: Immunoglobulin, A.

[21] Goldman A.S. (2000). Modulation of the gastrointestinal tract of infants by human milk. Interfaces and interactions. An evolutionary perspective. J. Nutr..

[22] Greco L., Auricchio S., Mayer M., Grimaldi M. (1988). Case control study on nutritional risk factors in celiac disease. J. Pediatr. Gastroenterol. Nutr..

[23] Lucas A., Cole T.J. (1990). Breast milk and neonatal necrotising enterocolitis. Lancet.

[24] Giromini C., Lovegrove J.A., Givens D.I., Rebucci R., Pinotti L., Maffioli E., Tedeschi G., Sundaram T.S., Baldi A. (2019). In vitro-digested milk proteins: Evaluation of angiotensin-1-converting enzyme inhibitory and antioxidant activities, peptidomic profile, and mucin gene expression in HT29-MTX cells. J. Dairy Sci..

[25] Baró L., Jiménez J., Martínez-Férez A., Bouza J. (2001). Bioactive milk peptides and proteins. Ars. Pharm..

[26] Haschke F., Haiden N., Thakkar S.K. (2016). Nutritive and bioactive proteins in breastmilk. Ann. Nutr. Metab..

[27] Lönnerdal B., Erdmann P., Thakkar S.K., Sauser J., Destaillats F. (2017). Longitudinal evolution of true protein, amino acids and bioactive proteins in breast milk: A developmental perspective. J. Nutr. Biochem..

[28] Mansour R.G., Stamper L., Jaeger F., McGuire E., Fouda G., Amos J., Barbas K., Ohashi T., Alam S.M., Erickson H. (2016). The presence and anti-HIV-1 function of tenascin C in breast milk and genital fluids. PLoS ONE.

[29] Charlwood J., Hanrahan S., Tyldesley R., Langridge J., Dwek M., Camilleri P. (2002). Use of proteomic methodology for the characterization of human milk fat globular membrane proteins. Anal. Biochem..

[30] Froehlich J.W., Dodds E.D., Barboza M., McJimpsey E.L., Seipert R.R., Francis J., An H.J., Freeman S., German J.B., Lebrilla C.B. (2010). Glycoprotein expression in human milk during lactation. J. Agric. Food Chem..

[31] Huang J., Kailemia M.J., Goonatilleke E., Parker E.A., Hong Q., Sabia R., Smilowitz J.T., German J.B., Lebrilla C.B. (2017). Quantitation of human milk proteins and their glycoforms using multiple reaction monitoring (MRM). Anal. Bioanal. Chem..

[32] Picariello G., Ferranti P., Mamone G., Roepstorff P., Addeo F. (2008). Identification of N-linked glycoproteins in human milk by hydrophilic interaction liquid chromatography and mass spectrometry. Proteomics.

[33] Zheng F., Du Y.M., Lin X.S., Zhou L.Q., Bai Y., Yu X.B., Voglmeir J., Liu L., Du Y., Lin X. (2019). N-Glycosylation Plays an Essential and Species-Specific Role in Anti-Infection Function of Milk Proteins Using Listeria monocytogenes as Model Pathogen. J. Agric. Food Chem..

[34] Alocci D., Mariethoz J., Gastaldello A., Gasteiger E., Karlsson N.G., Kolarich D., Packer N.H., Lisacek F. (2018). GlyConnect: Glycoproteomics Goes Visual, Interactive, and Analytical. J. Proteome Res..

[35] Picariello G., Ferranti P., Mamone G., Klouckova I., Mechref Y., Novotny M.V., Addeo F. (2012). Gel-free shotgun proteomic analysis of human milk. J. Chromatogr. A.

[36] Van Herwijnen M.J.C., Zonneveld M.I., Goerdayal S., ’t Hoen E.N.M.N., Garssen J., Stahl B., Altelaar A.F.M., Redegeld F.A., Wauben M.H.M. (2016). Comprehensive proteomic analysis of human milk-derived extracellular vesicles unveils a novel functional proteome distinct from other milk components. Mol. Cell. Proteom..

[37] Zhang Q., Cundiff J.K., Maria S.D., McMahon R.J., Woo J.G., Davidson B.S., Morrow A.L. (2013). Quantitative analysis of the human milk whey proteome reveals developing milk and mammary-gland functions across the first year of lactation. Proteomes.

[38] Mosca F., Giannì M.L. (2017). Human milk: Composition and health benefits. Pediatr. Med. Chir..

[39] Gendler S.J., Dermer G.B., Silverman L.M., Tökés Z.A. (1982). Synthesis of α1-antichymotrypsin and α1-acid glycoprotein by human breast epithelial cells. Cancer Res..

[40] Liao Y., Alvarado R., Phinney B., Lönnerdal B. (2011). Proteomic characterization of human milk whey proteins during a twelve-month lactation period. J. Proteome Res..

[41] Orczyk-Pawiłowicz M., Hirnle L., Berghausen-Mazur M., Kątnik-Prastowska I.M. (2014). Lactation stage-related expression of sialylated and fucosylated glycotopes of human milk α-1-acid glycoprotein. Breastfeed. Med..

[42] Ceciliani F., Pocacqua V., Provasi E., Comunian C., Bertolini A., Bronzo B., Moroni P., Sartorelli P. (2005). Identification of the bovine alpha1-acid glycoprotein in colostrum and milk. Vet. Res..

[43] Orczyk-Pawiłowicz M., Berghausen-Mazur M., Hirnle L., Kątnik-Prastowska I. (2015). O-glycosylation of α-1-acid glycoprotein of human milk is lactation stage related. Breastfeed. Med..

[44] Yu Y., Lasanajak Y., Song X., Hu L., Ramani S., Mickum M.L., Ashline D.J., Prasad B.V.V., Estes M.K., Reinhold V.N. (2014). Human milk contains novel glycans that are potential decoy receptors for neonatal rotaviruses. Mol. Cell. Proteom..

[45] Lindberg T., Skude G. (1982). Amylase in human milk. Pediatrics.

[46] Jones J.B., Mehta N.R., Hamosh M. (1982). α-amylase in preterm human milk. J. Pediatr. Gastroenterol. Nutr..

[47] Fridhandler L., Berk J.E., Montgomery K.A., Wong D. (1974). Column-chromatographic studies of isoamylases in human serum, urine, and milk. Clin. Chem..

[48] Hurley W.L., Theil P.K. (2011). Perspectives on immunoglobulins in colostrum and milk. Nutrients.

[49] Bera T.K., Zimonjic D.B., Popescu N.C., Sathyanarayana B.K., Kumar V., Lee B., Pastan I. (2002). POTE, a highly homologous gene family located on numerous chromosomes and expressed in prostate, ovary, testis, placenta, and prostate cancer. Proc. Natl. Acad. Sci. USA.

[50] Sharma A., Albahrani M., Zhang W., Kufel C.N., James S.R., Odunsi K., Klinkebiel D., Karpf A.R. (2019). Epigenetic activation of POTE genes in ovarian cancer. Epigenetics.

[51] Bera T.K., Fleur A.S., Lee Y., Kydd A., Hahn Y., Popescu N.C., Zimonjic D.B., Lee B., Pastan I. (2006). POTE paralogs are induced and differentially expressed in many cancers. Cancer Res..

[52] Liu X.F., Bera T.K., Liu L.J., Pastan I. (2009). A primate-specific POTE-actin fusion protein plays a role in apoptosis. Apoptosis.

[53] Kasahara Y., Osuka S., Takasaki N., Bayasula Koya Y., Nakanishi N., Murase T., Nakamura T., Goto M., Iwase A., Kajiyama H. (2021). Primate-specific POTE-actin gene could play a role in human folliculogenesis by controlling the proliferation of granulosa cells. Cell. Death Discov..

[54] Zhou S.M., Cheng L., Guo S.J., Wang Y., Czajkowsky D.M., Gao H., Hu X.F., Tao S.C. (2015). Lectin RCA-I specifically binds to metastasis-associated cell surface glycans in triple-negative breast cancer. Breast Cancer Res..

[55] Ryu K.Y., Sinnar S.A., Reinholdt L.G., Vaccari S., Hall S., Garcia M.A., Zaitseva T.S., Bouley D.M., Boekelheide K., Handel M.A. (2008). The mouse polyubiquitin gene Ubb is essential for meiotic progression. Mol. Cell. Biol..

[56] Ryu H.-W., Park C.-W., Ryu K.-Y. (2014). Disruption of polyubiquitin gene Ubb causes dysregulation of neural stem cell differentiation with premature gliogenesis. Sci. Rep..

[57] Ryu K.-Y., Garza J.C., Lu X.-Y., Barsh G.S., Kopito R.R. (2008). Hypothalamic neurodegeneration and adult-onset obesity in mice lacking the Ubb polyubiquitin gene. Proc. Natl. Acad. Sci. USA.

[58] Park C.W., Jung B.K., Ryu K.Y. (2020). Disruption of the polyubiquitin gene Ubb reduces the self-renewal capacity of neural stem cells. Biochem. Biophys Res Commun..

[59] Baker R.T., Board P.G. (1987). The human ubiquitin gene family: Structure of a gene and pseudogenes from the Ub B subfamily. Nucleic Acids Res..

[60] Liu L., Jiang L., Ding X.D., Liu J.F., Zhang Q. (2015). The regulation of glucose on milk fat synthesis is mediated by the ubiquitin-proteasome system in bovine mammary epithelial cells. Biochem. Biophys. Res. Commun..

