# Supplementary material for: Analysis of Minor Proteins Present in Breast Milk by Using WGA Lectin
Source: Children (Basel). 2022 Jul 20;9(7):1084. doi: 10.3390/children9071084 (PMC9318462; doi:10.3390/children9071084)
Supplement: Supplementary file 1 [file children-09-01084-s001.zip › children-1799200-supplementary.pdf]

Supplementary Table S1: Proteins identified by mass spectrometry in each of the samples. Protein names, Uniprot database access number, score (detection reliability) and coverage (% of protein sequence detected) are shown.

PROTEIN			WHOLE SERUM						WGA LECTIN					
			5 months of breastfeeding		12 months of breastfeeding		18 months of breastfeeding		5 months of breastfeeding		12 months of breastfeeding		18 months of breastfeeding	
	Name	Access number	Score	Cov	Score	Cov	Score	Cov	Score	Cov	Score	Cov	Score	Cov
1	Actin, cytoplasmic 1	P60709	43.68	6.40			62.03	14.40						
2	Alpha-1-acid glycoprotein 1	P02763							16.48	7.40	31.40	10.90		
3	Alpha-1-antichymotrypsin	P01011	144.10	23.80	94.06	32.60	145.95	29.00	71.69	28.70	63.41	10.80		
4	Alpha-1-antitrypsin	P01009	21.35	5.00			23.96	4.00						
5	Alpha-2-HS-glycoprotein	C9JV77	26.03	7.30										
6	Alpha-amylase 1	P04745							14.73	2.30			12.43	2.10
7	Alpha-lactalbumin	P00709	431.63	83.00	212.77	48.50	361.28	80.20	197.10	38.70	50.30	23.90	136.35	44.30
8	Alpha-N-acetylgalactosaminide alpha-2,6-sialyltransferase 1	G3XAD9					11.33	1.40	10.13	2.10			8.66	2.10
9	Alpha-S1-casein	P47710	268.07	58.30	85.67	27.60	204.62	47.50	179.59	48.10	108.53	30.20	104.35	25.90
10	Apolipoprotein A-I	P02647	291.41	68.50			139.53	39.70	27.29	13.40	11.67	4.10	10.51	4.10

11	Apolipoprotein A-II	P02652	75.10	42.00	24.51	17.00	35.36	22.00						
12	Apolipoprotein E	H0Y7L5	32.70	8.90			11.43	4.10						
13	Beta-1,4-galactosyltransferase 1	P15291	90.98	18.80			84.53	21.80	34.13	4.70	10.83	2.70		
14	Beta-2-microglobulin	P61769	40.41	31.90			58.64	28.70						
15	Beta-casein	P05814	251.88	91.50	131.82	60.10	209.01	90.70	192.96	83.10	119.64	53.50	102.99	34.50
16	Bile salt-activated lipase	P19835	528.67	34.50	246.12	24.90	468.65	35.50	617.27	43.80	504.34	37.10	385.73	34.50
17	Butyrophilin subfamily 1 member A1	Q13410	247.76	27.70	92.33	14.80	149.71	23.50	63.27	8.70	114.10	15.50	14.68	2.40
18	Carbonic anhydrase 6	P23280					12.92	2.90	32.00	12.30			8.82	2.90
19	C-C motif chemokine 28	D6RC73	8.00	18.70			22.33	18.70	14.86	18.70	13.41	18.70	17.72	18.70
20	Chordin-like protein 2	Q6WN34	109.76	25.10			73.30	16.00	98.07	29.10	16.45	16.40		
21	Clusterin	P10909	354.62	36.50	147.79	23.30	345.59	36.50	240.54	32.90	261.04	34.20	138.11	23.60
22	Complement C3	P01024	213.07	9.30	33.59	1.70	55.91	3.40						
23	Complement C4-B	P0C0L5	363.80	19.20	194.26	9.90	272.73	16.90			224.71	13.60		
24	Cystatin-C	P01034	73.91	34.90			39.97	23.20						
25	E3 ubiquitin-protein ligase MYCBP2	O75592											22.69	0.40
26	Elongation factor 1-alpha 1	P68104					26.30	7.90						

27	Endoplasmic reticulum chaperone BiP	P11021					40.99	5.30						
28	Ezrin	E7EQR4	9.42	1.50			12.48	1.10						
29	Fatty acid synthase	P49327	162.51	7.10	30.85	1.70	606.26	21.80	21.84	1.00	52.72	2.50	44.26	1.60
30	Fibroblast growth factor-binding protein 1	Q14512	17.67	6.40			13.63	6.40			12.66	3.80		
31	Follistatin-related protein 1	Q12841	61.36	14.20										
32	Galectin-3-binding protein	Q08380	24.39	5.10			48.94	7.50	14.13	2.90			12.76	9.70
33	Gelsolin	P06396			13.09	0.90	18.67	2.40						
34	Haptoglobin	P00738	33.70	8.10			26.27	7.10	92.19	27.00			10.70	4.20
35	Heat shock 70 kDa protein 1B	P0DMV9	18.61	1.70										
36	Hemopexin	P02790					14.92	5.10	10.51	2.80	32.70	9.70		
37	Hydroxyacyl-coenzyme A dehydrogenase, mitochondrial	Q16836	9.95	2.50	10.66	2.50	12.29	2.50			8.49	2.50		
38	Immunoglobulin heavy constant alpha 1	P01876	282.52	55.50	91.95	15.00	249.07	51.50	219.93	39.60	246.33	47.70	180.05	27.80
39	Immunoglobulin heavy constant gamma 1	P01857					29.97	7.00					14.24	4.00

40	Immunoglobulin heavy constant mu	P01871	17.36	5.00	29.37	5.00	22.06	5.00	11.06	1.60	16.46	4.60		
41	Immunoglobulin heavy variable 3/OR16-9 (non-functional)	A0A0B4J2B5							14.28	18.30	22.69	18.30	21.93	18.30
42	Immunoglobulin J chain	P01591	138.94	44.60	23.45	17.10	116.67	42.10	126.73	43.30	85.41	35.20	47.46	22.20
43	Immunoglobulin kappa constant	P01834			62.02	48.50	68.12	55.10	119.28	87.80	136.90	79.40	109.37	55.10
44	Immunoglobulin kappa variable 3-20	P01619							10.87	7.70	26.30	19.80		
45	Immunoglobulin kappa variable 3D-15	A0A087WSY6					9.52	6.00						
46	Immunoglobulin kappa variable 3D-20	A0A0C4DH25					8.37	6.00						
47	Immunoglobulin kappa variable 3D-7	A0A075B6H7							13.08	7.70				
48	Immunoglobulin lambda constant 2	P0DOY2	39.41	30.10	35.61	27.30	63.63	48.10	86.34	66.90			53.72	49.00
49	Immunoglobulin lambda-like polypeptide 5	B9A064			14.49	4.60					98.62	34.40		
50	Insulin-like growth factor-binding protein 2	P18065	53.16	12.60			34.18	10.70						
51	Kallikrein-6	Q92876					25.34	9.40						

52	Kappa-casein	P07498	153.59	69.70	113.70	54.30	139.05	67.50	140.07	56.00	70.21	32.40	100.96	40.60
53	Keratin, type I cytoskeletal 9	P35527					11.82	5.80	11.98	1.40			11.82	1.50
54	Keratin, type II cytoskeletal 1	P04264					60.94	6.60	68.52	9.00			109.73	12.40
55	Lactadherin	Q08431	36.81	8.70	17.70	3.20	39.31	10.40	65.94	15.10	82.90	21.20	41.28	10.40
56	Lactoperoxidase	P22079	38.78	4.00					12.52	0.80				
57	Lactotransferrin	P02788	1383.92	80.70	614.08	51.10	1164.42	80.20	962.86	68.30	612.07	55.80	427.74	38.30
58	Leucine-rich alpha-2-glycoprotein	P02750			31.64	12.10					14.69	4.60		
59	Lipoprotein lipase	P06858	142.43	19.70			45.89	7.10	143.82	29.20	34.46	7.10		
60	Lysozyme C	P61626	168.57	62.10	39.60	27.00	156.75	72.20	65.44	29.70	33.80	27.00	12.94	8.70
61	Macrophage mannose receiver 1	P22897	87.21	5.60			199.70	11.80	227.75	14.00	119.60	7.70	180.59	11.40
62	Metalloproteinase inhibitor 1	H0Y789	28.58	26.60			28.93	26.60						
63	Monocyte differentiation antigen CD14	P08571	204.81	44.00	57.70	13.00	247.50	49.80	93.60	23.70	110.24	23.40	33.38	17.30
64	Mucin-1	P15941	30.54	9.60	13.88	7.40	34.33	12.20			13.55	7.40		
65	Nucleobindin 2, isoform CRA_b	A0A087WSV8	60.67	19.70			79.91	16.40						
66	Nucleobindin-1	Q02818	56.74	10.40			42.67	8.40						
67	Osteopontin	P10451	137.27	49.60	147.59	40.70	293.25	63.60			90.57	23.20	90.62	23.20

68	Peptidyl-prolyl cis-trans isomerase B	P23284	28.42	10.60			27.32	10.10						
69	Perilipin-2	Q99541	21.17	4.30	31.28	5.00	11.02	2.90						
70	Plasma protease C1 inhibitor	P05155	18.05	1.80					71.01	14.60	41.26	6.80		
71	Plasma serine protease inhibitor	P05154			24.10	7.30								
72	Polymeric immunoglobulin receptor	P01833	620.30	42.50	300.22	25.90	716.26	52.00	510.02	38.00	444.13	36.10	365.48	30.30
73	Polyubiquitin-B	B4DV12									25.32	16.30		
74	POT ankyrin domain family member F	A5A3E0							11.77	1.00				
75	Prosaposin	C9JIZ6	27.82	5.50	29.78	5.50	38.93	7.20	8.30	8.30	52.60	4.90	13.84	8.30
76	Prothrombin	C9JV37	21.12	5.80										
77	Serotransferrin	P02787	87.52	11.00			104.77	13.70						
78	Serum albumin	P02768	745.51	71.20	89.89	9.60	588.59	58.10	72.45	9.60			89.09	12.20
79	Sulfhydryl oxidase 1	O00391	20.68	1.70	10.36	0.90	71.07	11.30	121.73	13.60	197.19	21.60	69.05	7.40
80	Transcobalamin-1	P20061	9.25	1.80	24.50	3.60	97.27	18.40						
81	UTP--glucose-1-phosphate uridylyltransferase	Q16851	9.91	1.70	11.43	3.70								

82	Vitamin D-binding protein	D6RF35	49.60	7.30	12.17	6.30	24.77	2.70				
83	Vitronectin	P04004	13.80	3.10			47.46	7.70			16.54	3.10
84	Xanthine dehydrogenase/oxidase	P47989			247.65	17.90	18.28	1.40			211.35	14.40
